# Critical appraisal and narrative review of the literature in IVF/ICSI patients with adenomyosis and endometriosis

**DOI:** 10.3389/frph.2024.1525705

**Published:** 2024-12-24

**Authors:** Ramazan Mercan, Can Benlioglu, Gulumser Ece Aksakal

**Affiliations:** ^1^ Department of Obstetrics and Gynecology, School of Medicine, Koc University, İstanbul, Türkiye; ^2^ Department of Obstetrics and Gynecology, American Hospital, İstanbul, Türkiye

**Keywords:** endometriosis, *in vitro* fertilization, coexisting endometriosis and adenomyosis, Ultralong GnRH suppression, frozen embryo transfer

## Abstract

Endometriosis and adenomyosis are prevalent causes of infertility, often coexisting in a significant proportion of patients. Although endometriosis typically does not negatively impact assisted reproductive technology (ART) outcomes, the presence of coexisting adenomyosis, mainly non-severe external forms, may slightly influence IVF/ICSI success rates. However, this impact is often minimal and may result in insignificant changes in statistical analyses. Recent studies underscore the critical role of accurate diagnostic techniques, such as ultrasound or MRI, in identifying severe adenomyosis characteristics, including diffuse involvement with junctional zone participation. This precise delineation is reassuring, as it is essential for tailoring assisted reproductive technology (ART) strategies to enhance success rates and reduce the confounding effects of adenomyosis, particularly when it coexists with endometriosis. Strategic approaches, such as ultralong GnRH agonist protocols or freeze-all strategies, may provide advantages in these scenarios. However, the need for extensive research is vital to understanding the complex interactions between endometriosis, adenomyosis, and ART outcomes. This ongoing exploration is particularly important in cases where coexisting adenomyosis might not significantly influence statistical results.

## Introduction

Endometriosis and adenomyosis can cause infertility and often present together, with co-occurrence rates approaching 90%. This high coexistence rate underscores the complexity and necessitates further research (1). Both conditions share evolutionary theories and pathophysiological mechanisms, including Müllerian metaplasia, stem cell differentiation and genetic alterations, elevated inflammatory markers. Despite their shared characteristics, the published data about the impact of concurrent endometriosis and adenomyosis on assisted reproductive technology (ART) outcomes is limited.

We critically appraise the current literature, primarily focusing on meta-analyses and large cohort studies, to evaluate the impacts of adenomyosis and endometriosis on ART outcomes, particularly in cases where they occur concurrently. By scrutinizing the data, we aim to identify scientific pitfalls and challenges in the existing studies, emphasizing the high concordance rate between these two conditions and their potential impact on reported outcomes. Additionally, we explore various mitigation strategies, including surgery, optimal ovarian stimulation protocols, and frozen embryo transfer (FET) efficacy in affected patients.

## Endometriosis

### Definition, pathophysiology, prevalence, clinical manifestations and rationale behind the infertility

Endometriosis is defined by endometrial glands and stroma external to the uterus and impacts approximately 6%–8% of the general population and 20%–30% of women experiencing subfertility (1). Classification is typically based on the anatomical location of the lesions, including peritoneal, ovarian, or deep infiltrating endometriosis, with patients presenting multiple sites concurrently (2). Clinical manifestations include chronic pelvic pain, dysmenorrhea, dyspareunia, dyschezia, and infertility. While the precise etiology of endometriosis remains vague, several etiopathogenetic mechanisms have been hypothesized, including retrograde menstruation, Müllerian metaplasia, stem cell differentiation, lymphatic dissemination, and epigenetic modifications.

Endometriosis is highly prevalent among subfertilite population, but despite this correlation, the precise pathophysiological mechanisms underlying this association remain inconclusive, aside from tubal blockage resulting from adhesion formation. Several ideas have been proposed, including reduced oocyte quality, impaired endometrial receptivity, elevated levels of inflammatory markers, cytokines, and growth factors, overexpression of P450 enzymes, oxidative stress, prostaglandin imbalances, and the presence of coexisting conditions such as adenomyosis.

The ambiguous association between endometriosis and subfertility can also be seen in studies showing IVF/ICSI success rates of endometriosis patients. For example, in Horton et al.'s meta-analysis of 29 studies, the authors found a 15% reduction in conception likelihood and a 12% reduction in live birth rates (LBR) after IVF/ICSI in women with endometriosis compared to those without (3). However, these effects either lost their significance in subgroup analyses or, in the case of deep infiltrating endometriosis (DIE), the population could not be included due to increased heterogeneity among the studies. Moreover, Santully et al. claimed that endometrioma *per se* is not a cause of infertility, as the authors showed similar euploid blastocyst rates and outcomes after euploid embryo transfer in patients with and without endometriosis, indicating neither oocyte quality nor endometrial receptivity is impaired (2). Similar findings were also demonstrated in two recent large cohort studies (4, 5). Moreover, Qu et al.'s meta-analysis highlighted lower oocyte numbers and implantation rates in women with endometriosis but no difference in overall reproductive outcomes (6).

Overall, endometriosis might impact specific aspects of the IVF/ICSI process (like the number of oocytes retrieved and the rates of cycle cancellation); however, reproductive outcomes, including the success rates of euploid embryo transfers, are not significantly different from those observed in patients without endometriosis.

### The impact of surgical treatment on IVF/ICSI outcomes

A meta-analysis by Hamdan et al. determined that IVF/ICSI outcomes are comparable between patients with and without endometrioma, with a higher cycle cancellation rate in the endometriosis group. Additionally, the authors concluded that surgical treatment does not alter outcomes in patients with endometrioma (7). Similarly, another meta-analysis conducted by Wu et al. found that surgical treatment of endometrioma prior to IVF has no significant effect on the number of mature oocytes or live birth rates. Nonetheless, the total number of oocytes retrieved was lower in the surgical treatment group (8). Consistent findings were reported in another Alshehre et al. meta-analysis, and they demonstrated a decrease in the number of oocytes and metaphase II (MII) oocytes in the surgical treatment group. Agreeing with the current literature, they found no significant differences in gonadotropin dose, duration of stimulation, total number of embryos, number of high-quality embryos, clinical pregnancy rates (CPR), implantation rates (IR), or live birth rates (LBR) (9).

### Ultralong GnRH suppression and frozen embryo transfer (FET) in IVF/ICSI outcomes

A meta-analysis by Sallam et al. (10) indicated an increase in clinical pregnancy rates with ultralong GnRH agonist treatment. However, a more recent Cochrane review found no significant difference in outcomes between ultralong GnRH suppression and other treatment modalities (11).

The effectiveness of frozen embryo transfer (FET) in patients with endometriosis remains a topic of debate. Bourdon et al., in a retrospective study, observed higher cumulative pregnancy rates in patients with endometriosis undergoing FET compared to those undergoing fresh embryo transfer (12). Similarly, Wu et al., in a retrospective study involving 506 frozen and 255 fresh embryo transfers in patients with advanced endometriosis, reported better pregnancy and neonatal outcomes with FET than fresh embryo transfer (13). Conversely, Tan et al., in a retrospective cohort study, found no significant difference in early pregnancy outcomes between fresh and FET cycles in patients with endometriosis (14). In a recent meta-analysis, Paffoni et al. reported a modest decrease in live birth rates in patients with endometriosis. However, this difference was not statistically significant after adjusting for confounding factors (15).

## Adenomyosis

### Definition, pathophysiology, prevalence, clinical manifestations and rationale behind the infertility

Adenomyosis is the presence of endometrial glands and stroma within the myometrium of the uterus. Several pathophysiological mechanisms have been submitted, including invagination of the basal endometrium, Müllerian metaplasia, stem cell differentiation and epigenetic changes. It is also hypothesized that external adenomyosis may develop secondary to deep infiltrating endometriosis and is more frequent in patients with primary infertility and younger patients (16–18). In contrast, intrinsic adenomyosis could be associated with invasive interventions to the endometrium (like multiple curettages) and older age (19).

The prevalence of adenomyosis varies significantly based on age and diagnostic methods. Reports indicate a prevalence of 24.4% in women aged 40 and older and 7.52% in women younger than 40 (20, 21). Overall, prevalence ranges from 5% to 70% but generally stands around 10% for isolated adenomyosis, 1% for adenomyosis with coexisting fibroids, 6% for adenomyosis coexisting with endometriosis, and 7% for adenomyosis coexisting with both fibroids and endometriosis in women with subfertility (22). The increasing prevalence is attributed to advancements in ultrasound resolution and magnetic resonance imaging (MRI) for diagnosis (20). Classification of adenomyosis varies, often distinguishing between external and intrinsic types. Interestingly, symptoms do not significantly differ between these classifications (23).

Several theories have been proposed regarding the etiology of infertility in patients with adenomyosis, including increased uterotubal peristalsis affecting gamete transport and altered adhesion molecules (HOXA10, FOXO1A, leukemia inhibiting factor, integrin-beta-3 and osteopontin levels) (24, 25). Moreover, in a multicenter cohort study, transcriptomic analysis of the endometrium from patients with adenomyosis revealed a higher prevalence of a non-receptive endometrial profile compared to controls, suggesting that molecular changes in the endometrium may compromise endometrial receptivity (26). In a recent systemic review and meta-analysis, retrograde uterine contraction frequency was found to be increased in patients with endometriosis and adenomyosis, which may also contribute to menstrual pain and infertility (27)

Numerous published results on the impact of ART outcomes on patients with adenomyosis yield heterogeneous findings. Although the studies with euploid and donor cycles have shown similar pregnancy and live birth rates compared to those without adenomyosis (28, 29), several observational studies and meta-analyses demonstrated escalating trends in adverse ART outcomes. For instance, three meta-analyses showed lower clinical pregnancy and live birth rates and increased miscarriage rates in patients with adenomyosis (30–32). One of these studies, Nirgianakis et al., demonstrated a significant decrease in clinical pregnancy and live birth rates in patients with adenomyosis (33). However, the decrease in live birth rates became insignificant when adjusted for age, similar to euploid transfer and donor cycles mitigating female age's impact.

However, miscarriage rates present a different picture, with multiple studies having noted significantly higher miscarriage rates in patients with adenomyosis. This trend is particularly pronounced when adenomyosis is in the junctional zone (JZ), during donor oocyte cycles, and following euploid embryo transfers (34–36). Additionally, diffuse adenomyosis has been associated with decreased pregnancy rates and higher miscarriage rates, indicating a more adverse impact on reproductive outcomes in these cases (37).

In addition to the negative impact on ART outcomes, pregnancy complications are increased in patients with adenomyosis. Observational and meta-analysis have shown higher risks of preterm delivery, preterm premature rupture of membranes, and preeclampsia in patients with adenomyosis (38).

### Ultralong GnRH suppression and frozen embryo transfer (FET) in IVF/ICSI outcomes

Ultralong GnRH agonist suppression in IVF/ICSI treatment aims to suppress the growth of ectopic endometrial tissue, decrease inflammatory markers, and reduce uterine size, theoretically improving treatment outcomes. The effectiveness of extended GnRH agonist suppression on IVF/ICSI outcomes remains controversial. For instance, two observational studies reported increased pregnancy rates following 1–3 months of GnRH agonist suppression (39, 40) and one retrospective study with 374 adenomyosis patients did not find a significant difference in IVF/ICSI outcomes with extended GnRH agonist suppression (41). In a study with propensity-score matching analysis, the authors found no significant difference in live birth or cumulative live birth rates between patients undergoing a GnRH-antagonist protocol with a freeze-all strategy and those using a long-acting GnRH agonist protocol in women with adenomyosis (42).

## Coexisting endometriosis and adenomyosis

Coexistence of endometriosis with adenomyosis is prevalent, found in 65% to 90% of patients (43–45). Additionally, these pathologies share common pathophysiological features and similar genetic mutations like KRAS (46). Both conditions exhibit increased aromatase activity, leading to excessive estrogen production, up-regulation of inflammatory cytokines, and expression of cyclooxygenase-2, resulting in increased levels of prostaglandin-E2 and, ultimately, progesterone resistance (46).

There is limited data available on assisted reproductive technology (ART) outcomes in patients with coexisting endometriosis and adenomyosis. Two studies reported lower pregnancy rates in patients with coexistent endometriosis and adenomyosis compared to those with endometriosis alone (47, 48). Sharma et al. specifically noted significantly reduced pregnancy rates in patients with both conditions compared to those with endometriosis alone. However, there is no difference compared to patients with adenomyosis alone, suggesting that adenomyosis may primarily contribute to the decreased pregnancy rate (46). Another study by Shi et al., which included 176 patients with coexistent endometriosis and adenomyosis, revealed that patients who achieved live birth had smaller endometrioma and uterine sizes, indicating that anatomical distortion and diffuse adenomyosis might play a more critical role than adenomyosis alone (49).

Rees et al. (50) found that patients with coexisting endometriosis and adenomyosis had lower pregnancy rates compared to those with either endometriosis or adenomyosis alone. In a recent systematic review, Wang et al. reported that pregnancy rates were lower and miscarriage rates were higher in patients with adenomyosis, particularly in cases of diffuse adenomyosis, but not in asymptomatic cases (51). Additionally, coexistence with fibroids is more prevalent in internal adenomyosis than external forms, potentially resulting in lower pregnancy rates (18% vs. 2.8%) (52). It' s coherent with a recent study which demonstrated that patients with internal adenomyosis were typically older and more frequently had associated fibroids compared to those with external adenomyosis (53).

Kishi et al. reported that up to 96% of patients with adenomyosis in the outer myometrium had coexisting deep endometriosis (DE), contrasting with only 15% of those with inner myometrium adenomyosis (18).

## Summary of findings

### Endometriosis

Based on current findings, it is reasonable to conclude that *in vitro* fertilization (IVF) outcomes are not impaired in patients with endometriosis. Despite theoretical concerns about decreased endometrial receptivity and increased inflammatory markers, donor transfer cycles' data show similar pregnancy rates in those patients. Overall, studies indicate that surgical treatment before assisted reproductive technology (ART) does not improve outcomes. It can reduce ovarian reserve and the number of oocytes retrieved, potentially affecting cumulative live birth rates. While there are promising results for FET in patients with endometriosis, further studies are required to clarify its role. Some studies have reported improved pregnancy and neonatal outcomes with FET compared to fresh embryo transfer, while others have found no significant difference.

### Adenomyosis

The overall evidence indicates that adenomyosis negatively impacts outcomes in assisted reproductive technology. While there are conflicting findings regarding pregnancy rates, patients with adenomyosis consistently exhibit higher miscarriage rates. Notably, IVF/ICSI outcomes do not differ significantly in patients with asymptomatic or focal adenomyosis compared to those without adenomyosis. However, diffuse adenomyosis and involvement of the junctional zone are associated with poorer outcomes in IVF/ICSI. For women with adenomyosis undergoing ART treatment, strategies such as a freeze-all approach or ultralong GnRH agonist protocols may be advantageous. Although progesterone resistance is acknowledged as a significant factor in infertility seen in patients with both endometriosis and adenomyosis, increasing progesterone doses for luteal support does not appear to enhance pregnancy rates, and there was no significant difference in progesterone levels between patients who achieved pregnancy and those who did not. These findings emphasize the multifactorial nature of infertility in adenomyosis and the need for comprehensive management strategies that consider both hormonal and anatomical aspects to optimize fertility outcomes in affected patients.

### Adenomyosis as a confounding factor for infertile patients with endometriosis

These findings suggest that,

• Endometriosis typically involves external forms that do not significantly impact pregnancy and miscarriage rates.
• Patients with both endometriosis and severe or diffuse adenomyosis constitute a small subset of those with endometriosis, and even if they experience lower pregnancy rates within this group, their impact on overall results is minimal and does not change final statistical analysis.
• Patients with endometriosis coexisting with diffuse and severe adenomyosis that may impair ART outcomes are more likely prioritized as adenomyosis rather than endometriosis.

## Conclusion

In conclusion, current findings indicate that IVF/ICSI outcomes do not differ between patients with endometriosis and those without. Although adenomyosis coexists in a majority of patients with endometriosis, it typically presents as external adenomyosis, which does not affect uterine anatomy and plays a minimal role in outcomes. The coexistence of endometriosis and internal adenomyosis is relatively rare. Although this subgroup exhibits lower live birth rates and higher miscarriage rates, its rarity often causes it to blend into broader endometriosis studies, preventing statistical significance in overall analyses unless a prospective study specifically focuses on these concurrent conditions.

Yet the sheer number of published studies for both pathologies, a skeptical approach is a must, considering the scarcity of concurrent studies and the fact that the published studies do not address these intertwined pathologies separately.

## Data Availability

The original contributions presented in the study are included in the article/Supplementary Material, further inquiries can be directed to the corresponding author.
